# Sex differences in circumstances and consequences of outdoor and indoor falls in older adults in the MOBILIZE Boston cohort study

**DOI:** 10.1186/1471-2318-13-133

**Published:** 2013-12-06

**Authors:** Rachel L Duckham, Elizabeth Procter-Gray, Marian T Hannan, Suzanne G Leveille, Lewis A Lipsitz, Wenjun Li

**Affiliations:** 1 Health Statistics and Geography Lab, Division of Preventive and Behavioral Medicine, University of Massachusetts Medical School, Shaw SH2-230, 55 Lake Avenue North, Worcester, MA, USA; 2 Institute for Aging Research, Hebrew SeniorLife /Harvard Medical School, and Beth Israel Deaconess Medical Center, 1200 Centre Street, Boston, MA 02131-1097, USA; 3 College of Nursing and Health Sciences, University of Massachusetts, 100 Morrissey Blvd, Boston, MA 02125-3393, USA

**Keywords:** Falls, Injury, Aging, Physical activity, Space use, Sex, Gender

## Abstract

**Background:**

Despite extensive research on risk factors associated with falling in older adults, and current fall prevention interventions focusing on modifiable risk factors, there is a lack of detailed accounts of sex differences in risk factors, circumstances and consequences of falls in the literature. We examined the circumstances, consequences and resulting injuries of indoor and outdoor falls according to sex in a population study of older adults.

**Methods:**

Men and women 65 years and older (N = 743) were followed for fall events from the Maintenance of Balance, Independent Living, Intellect, and Zest in the Elderly (MOBILIZE) Boston prospective cohort study. Baseline measurements were collected by comprehensive clinical assessments, home visits and questionnaires. During the follow-up (median = 2.9 years), participants recorded daily fall occurrences on a monthly calendar, and fall circumstances were determined by a telephone interview. Falls were categorized by activity and place of falling. Circumstance-specific annualized fall rates were calculated and compared between men and women using negative binomial regression models.

**Results:**

Women had lower rates of outdoor falls overall (Crude Rate Ratio (RR): 0.72, 95% Confidence Interval (CI): 0.56-0.92), in locations of recreation (RR: 0.34, 95% CI: 0.17-0.70), during vigorous activity (RR: 0.38, 95% CI: 0.18-0.81) and on snowy or icy surfaces (RR: 0.55, 95% CI: 0.36-0.86) compared to men. Women and men did not differ significantly in their rates of falls outdoors on sidewalks, streets, and curbs, and during walking. Compared to men, women had greater fall rates in the kitchen (RR: 1.88, 95% CI: 1.04-3.40) and while performing household activities (RR: 3.68, 95% CI: 1.50-8.98). The injurious outdoor fall rates were equivalent in both sexes. Women’s overall rate of injurious indoor falls was nearly twice that of men’s (RR: 1.98, 95% CI: 1.44-2.72), especially in the kitchen (RR: 6.83, 95% CI: 2.05-22.79), their own home (RR: 1.84, 95% CI: 1.30-2.59) and another residential home (RR: 4.65, 95% CI: 1.05-20.66) or other buildings (RR: 2.29, 95% CI: 1.18-4.44).

**Conclusions:**

Significant sex differences exist in the circumstances and injury potential when older adults fall indoors and outdoors, highlighting a need for focused prevention strategies for men and women.

## Background

Falls in the older population are a major health concern and the leading cause of unintentional fatal and non-fatal injuries in those 65 years and older [1,2]. In the United States approximately 30% of people over the age of 65 and 50% of people over the age of 80 years fall each year with approximately 10% of falls resulting in serious injury [1,3-5]. The growing economic burden of falls and fall-related injuries in the United States amounts to $20 billion (in 2007 dollars) each year, and is projected to increase to $54.9 billion by 2020 [6-8]. In addition to the cost and consequences, falls negatively impact an individual’s health, behavior and quality of life by increasing a fear of falling. The fear of falling may trigger a restriction in activity, decreased mobility, increased social isolation and a loss of independence [3,9].

Most falls in the older population are results of an inter-play of predisposing and precipitating factors. The predisposing factors previously identified have included increasing age, sex, age-associated changes in strength and balance, sensory impairments and chronic disease. The precipitating factors include acute illness, medications, urinary tract symptoms, hypotension, and muscle weakness [10]. Risk for falling may be further exacerbated by poor footwear, and environmental factors such as wet floors, outdoor weather conditions and poor lighting [11]. Since older men and women may differ in behaviors, prevalence of fall-related medical conditions and physical functions, risk factors as well as fall injuries likely differ by sex. For instance, women are believed to experience a greater number of falls and higher risk of injury from falling due to poorer lower extremity strength, more difficulties with activities of daily living and higher prevalence of osteoporosis, making them more susceptible to fracture compared to men [12].

Our previous studies have indicated the importance of determining risk factors, circumstances and consequences of indoor and outdoor falls independently [13] when falls prevention programs are formulated. Furthermore, our work [13] indicates that contrary to previous beliefs falls are not necessarily a marker of poor health and falling outdoors can occur as often as falling indoors and may be attributed to an active lifestyle.

Despite the extensive epidemiological research on risk factors associated with falling [3-5,10,12], and the current interventions for fall prevention focusing on modifiable factors, there is a lack of detailed information on sex-specific circumstances and consequences of falls in older adults [11,14,15], especially related to outdoor falls [16-18]. The objective of this study therefore was to examine the sex specific differences in health and behaviors attributing to the circumstances and injuries of falls in older adults.

## Methods

### Study design and participants

Community-dwelling older men (n = 276) and women (n = 467) were followed for fall events by the Maintenance of Balance, Independent Living, Intellect, and Zest in the Elderly (MOBILIZE) Boston study, a prospective cohort design, which has been described in detail elsewhere [19]. In brief, between September 2005 and December 2007, 749 (473 women) community-dwelling older adults, aged 70 years and older were recruited to examine novel risk factors and mechanisms of falls in older adults. Participant eligibility criteria included the ability to read and speak English, adequate cognition (18 points or more scored on the Mini-Mental State Examination (MMSE)), ability to walk 20-feet without assistance, and residing in the Boston, Massachusetts area for at least 2 years. For this analysis, 6 women over the age of 92 years were excluded to equalize the age ranges of men and women participants and minimize the potential differences in activities in non-overlapping ages by sex. Falls were recorded during a total of 2066.5 person-years of follow-up (September 2005-December 2009) and the median length of follow-up per participant was 2.9 years (range 0.04 to 4.3). Written informed consent was obtained from study participants. The Institutional Review Board of Hebrew Senior Life approved this study.

### Data collection

At baseline comprehensive assessments were completed during a clinical examination and home interview visit, and via self-administered questionnaires.

#### *Clinical examination*

During the clinical examination anthropometric measurements of height (measured by a stadiometer) and weight (measured with a balance beam scale) were assessed for each participant. Body Mass Index (BMI, kg/m^2^) was calculated from the weight in kilograms divided by height squared. Balance was measured with the Berg Balance Test [20], a multi-component assessment of standing balance. It consists of 14 subtests scored on a five-point scale (0-4), according to the quality or time taken to complete the task. The maximum score for the assessment is 56; a person with a score below 45 was considered as having balance deficit and an increased risk for falling [13]. An inability to perform chair stands was used as an indicator of poor lower extremity muscle strength. Gait Speed was the shortest time taken to complete a usual-paced 4-meter walk measured in meters per second (m/sec). A vision test to determine distance vision was completed from 10-feet; poor vision was defined as 40/100 or worse.

#### *Home interview visit*

During the home visits, trained interviewers administered questionnaires to determine the participant’s health and functionality, including questions on chronic diseases [21], health behaviors [22], fall history in the 12 months prior to baseline, medication adherence, and socio-demographic characteristics. As previously described [23], Activities of Daily Living (ADL) were scored on the ability of the participant to perform five activities (transferring, bathing, dressing, toileting, and eating). The number of comorbid conditions was determined by self-report in response to a query on *whether a health care provider had told them they have any of several medical conditions *[24]. Medication use was reported as the number of over the counter and prescription medications used in the past two weeks. Psychotropic medications (including the use of antidepressants, hypnotics, benzodiazepines, antipsychotics, and other sedatives) were classified separately. Fear of falling and cognitive function were measured using the Falls Efficacy Scale [25] and the Mini-Mental State Exam (MMSE), respectively. Health indicators were determined at the end of the home visits by asking participants to self-rate their health (excellent, good, fair or poor) and complete a Physical Activity Scale for the Elderly (PASE) [26], to assess the level of physical activity in the previous week. Participants were also asked specific questions regarding their walking habits. Participants were asked to record if and why they walked (never, for exercise, for utilitarian, both exercise and utilitarian), the number of city blocks walked per week (12 blocks = 1 mile) and the location in which they walked.

### Ascertainment of falls and fall circumstances

During the 4.3-year follow-up participants were instructed during the home visit to keep a daily falls calendar, which was mailed to the study staff on a monthly basis. For the falls calendar a fall was defined to the participant as being an event that resulted in unintentionally coming to rest on the ground or a lower surface. For participants who reported a fall, study staff would conduct a further telephone interview to ascertain the circumstances of the fall. Participants were asked to explain: 1) what happened when they fell on (date), 2) what they were doing when they fell, 3) where they were when they fell, and 4) the condition of the fall surface (e.g., dry vs. wet, hard vs. soft). For this analysis indoor and outdoor falls were grouped separately by the place of the fall, activity of the fall and the environment of the fall based on previous work from our group [23].

### Injurious falls

Falls were reported as causing injury if a participant self-reported being hurt in any way as a result of falling. These injurious falls were further classified as *bone fracture* and “*other serious injuries*” that included sprains, pulled or torn muscles, tendons or ligaments, dislocated joints, and concussions. In this analysis, a fall resulting in an overnight stay in a hospital regardless of injury was considered as a “*hospitalized*” fall.

### Statistical analysis

Descriptive statistics (mean, standard deviation and percentages) were used to characterize the cohort. As some of the data was not normally distributed non-parametric Wilcoxon rank-sum tests and Chi Squared tests were used to assess the differences in baseline characteristics between men and women. The rates of the place and activity-specific falls and injurious falls were computed and compared by sex using negative binomial regression models that account for over dispersion. Rates of falls are reported as number of falls per 1,000 person years. Women-to-men Rate Ratios (RR, the women’s rate divided by the men’s rate) and 95% confidence intervals (CI) were also computed using negative binomial regression models. An alpha level set at p < 0.05 was considered statistically significant. Both the adjusted and crude rate ratios were estimated for discussion purposes. First, we fit negative binomial regression models for indoor, outdoor and total falls using all of the following characteristics (excluding sex): age, education, race, body mass index, physical activity, blocks walked per week, outdoor walking type, balance, chair-stand ability, gait speed, activities of daily living, short physical performance battery, reduced activity due to illness in the past year, poor vision, bodily pain, number of comorbidities, self-rated health, peripheral neuropathy, foot pain, knee osteoarthritis, depression, number of medications, use of psychotropic medication, MMSE, Trails-B test, number of falls in year before baseline and falls efficacy. We calculated each person’s composite risk score as the summation of the products of the person’s risk level multiplied by the regression coefficient of the corresponding risk factor, for all risk factors included in the model. Then adjusted women/men rate ratios were calculated by including the appropriate composite risk score as a single covariate in the negative binomial model. All statistical analyses were performed with the statistical package STATA (StataCorp 2011. Stata Statistical Software: Release 12. College Station, Texas: STATACorp LP).

## Results

Baseline characteristics for men and women are presented in Table 1. In this analysis the age range of men and women was made equivalent, and mean (SD) of age at baseline were similar for men and women (78.3 ± 5.2 vs. 78.1 ± 5.0, p = 0.76). Compared to women, men in general appeared to be more physically fit, recording a faster gait speed (0.99 vs. 0.92 m/s, p = 0.002) and reporting more city blocks walked per week (53.8 vs. 36.3, p < 0.001), although men and women did not significantly differ in total physical activity in the past week assessed with the PASE questionnaire (*p = 0.44*). A significantly greater proportion of women reported doing light household chores in the past week (98.0% vs. 84.2% of men, p < 0.001), and a smaller percentage of women engaged in garden chores (32.0% vs. 41.2%, p = 0.01) in the past week. Men tended to report less daily body pain (30.9% vs. 44.2%, p < 0.001), foot pain (19.6% vs. 26.8%, p = 0.03), and psychotropic medication use (15.3% vs. 23.4%, p = 0.009).

**Table 1:** Baseline characteristics of men and women in the MOBILIZE Boston cohort

**Characteristics**	**Men**	**Women**	**p-value**^**†**^
	**(n = 276)**	**(n = 467)**	
**Demographic**			
Age (years)	78.3 ± 5.2	78.1 ± 5.0	*0.76*
Education completed (years)	14.8 ± 3.2	13.9 ± 3.0	*<0.001*
White race/ethnicity	227 (82.3)	351 (75.3)	*0.03*
**Lifestyle**			
Body mass index (kg/m^2^)	27.4 ± 4.4	27.7 ± 5.6	*0.67*
Physical activity (PASE)			
Moderate-vigorous occupational	21 (7.6)	56 (12.0)	*0.06*
Recreational (hours/week)	1.3 ± 2.4	0.9 ± 1.7	*0.07*
Light household chores in past week	229 (84.2)	431 (98.0)	*<0.001*
Lawn/garden chores in past week	113 (41.2)	148 (32.0)	*0.01*
Total PASE score	112.9 ± 79.6	104.2 ± 65.4	*0.44*
Blocks walked per week	53.8 ± 82.5	36.3 ± 57.7	*<0.001*
Outdoor walking habit			*0.55*
Non-walker	74 (27.3)	145 (31.7)	
Exercise only	66 (24.4)	111 (24.3)	
Utilitarian only	33 (12.2)	56 (12.3)	
Both exercise and utilitarian	98 (36.2)	145 (31.7)	
**Physical disability**			
Balance (Berg score)	50.5 ± 6.3	49.2 ± 6.9	*<0.001*
Assisted chair-stand test using arms (seconds)	19 (6.9)	39 (8.4)	*0.47*
Gait speed (m/sec)	0.99 ± 0.26	0.92 ± 0.25	*0.002*
Activities of daily living:			*0.71*
No difficulty	218 (79.0)	357 (76.5)	
Little/some difficulty	39 (14.1)	72 (15.4)	
Much difficulty/inability	19 (6.9)	38 (8.1)	
Short physical performance battery (score)	9.6 ± 2.4	9.1 ± 2.6	*0.01*
Reduced activity due to illness in past year	67 (24.3)	144 (30.8)	*0.06*
Poor vision (worse than 40/100)	21 (7.6)	39 (8.4)	*0.72*
**Illness-Related**			
Moderate/severe bodily pain	85 (30.9)	206 (44.2)	*<0.001*
Number of comorbid conditions	2.9 ± 1.6	2.9 ± 1.4	*0.74*
Fair/poor self-rated health	44 (15.9)	67 (14.4)	*0.56*
Peripheral neuropathy	44 (16.1)	47 (10.1)	*0.02*
Foot pain on most days	54 (19.6)	125 (26.8)	*0.03*
Knee osteoarthritis	67 (24.3)	122 (26.2)	*0.57*
Depression	22 (8.0)	31 (6.6)	*0.50*
Medication use			
Number of medications	6.2 ± 3.2	6.2 ± 3.1	*0.91*
Use of psychotropic medication^‡^	42 (15.3)	108 (23.4)	*0.009*
**Cognition**			
MMSE score	27.4 ± 2.5	26.8 ± 2.7	*0.004*
Trail making Test B adjusted for motor component (secs)	86.2 ± 61.9	91.5 ± 64.9	*0.35*
**Fall-Related**			
Number of falls in year before baseline	0.9 ± 3.3	0.7 ± 1.6	*0.17*
Falls efficacy score	96.3 ± 8.6	95.2 ± 9.7	*0.03*

All variables had a sample size of 95-100% of full N shown. Results are means ± SD or frequency (%) and corresponding *p-values* for sex differences.

^†^Wilcoxon rank-sum test was used for age, education, body mass index, PASE, balance, gait speed, short physical performance battery, number of comorbid conditions, number of medications, MMSE, Trail B, falls before baseline, falls efficacy, and blocks walked per week; chi-square was used for all other variables.

^‡^Includes antidepressants, benzodiazepines, antipsychotics, and other sedatives.

*Abbreviations:SD* Standard deviation, *PASE* Physical Activity Scale for the Elderly, *MMSE* Mini-Mental State Examination.

### Rates of all falls and circumstances

Over the 4.3-year study, 97% of the participants had 6 or more months of follow-up, and 93% of participants completed falls calendars for at least 1 year of follow-up. Major reasons for dropout included mortality/terminal illness and relocation out of the study area.

A total of 1680 falls (786 outdoors, 894 indoors) were reported during the 4.3-years follow-up (663 for men and 1017 for women). Men and women had comparable median lengths of follow-up time (2.8 vs. 2.9 years; *p = 0.42*), and percentages of persons reporting no falls (37.3% vs. 36.4%), one fall (20.3% vs. 20.8%), two falls (13.8% vs. 13.1%), and three or more falls (28.6% vs. 29.7%).

Although men and women did not differ in their rates of indoor falls (crude women-to-men RR: 1.05, 95% CI: 0.82-1.34), women reported significantly lower fall rates outdoors compared to men (0.72, 0.56-0.92), both inside (0.74, 0.56-0.98) and outside (0.68, 0.48-0.96) their own neighborhood. Lower fall rates in women were most notable in specific outdoor locations of recreation, such as on the golf course, and in forests and park areas (0.34, 0.17-0.70), just outside their home (0.65, 0.45-0.93) and during vigorous activity, such as hiking, tennis, and jogging (0.38, 0.18-0.81) compared to men. Women were less likely to fall on snowy or icy surfaces (0.55, 0.36-0.86) compared with men. In contrast, there were no sex differences in rates of falling outdoors on sidewalks, streets, and curbs (1.03, 0.75-1.41), and during walking activities (0.83, 0.61-1.130). All other outdoor circumstances of falls were not significantly different in women and men (Table 2). Indoors, women had a lower rate of falls on stairs (0.57, 0.34-0.95), but had greater fall rates in the kitchen (1.88, 1.04-3.40), and while performing household activities (3.68, 1.50-8.98) compared to men. Women also had higher rates of falls in the bathroom and in other peoples’ homes, but these differences were not statistically significant (1.63, 0.79-3.37 and 1.80, 0.84-3.87, respectively).

**Table 2:** Place and activity-specific numbers, rates and women/men rate ratios (95% Confidence Intervals) of falls and injurious falls by sex (per 1,000 person-years)

			**All falls**					**Falls resulting in injury**		
	**Number of falls**^**#**^	**Men’s rate**	**Women’s rate**	**Crude rate ratio**	**Adjusted†**	**Number of falls**^**#**^	**Men’s rate**	**Women’s rate**	**Crude rate ratio**	**Adjusted†**
				**(Women/Men)**	**Rate ratio**				**(Women/Men)**	**Rate ratio**
					**(Women/Men)**					**(Women/Men)**
**Total falls**	1680	915	779	0.85 (0.70 - 1.04)	1.01 (0.84 - 1.21)	715	281	385	1.37 (1.09 - 1.70)**	1.45 (1.17 - 1.80)**
**Total outdoor falls**	786	461	330	0.72 (0.56 - 0.92)**	0.84 (0.67 - 1.05)	364	174	176	1.01 (0.77 - 1.33)	1.15 (0.88 - 1.51)
**I. Place of outdoor fall**										
In own neighborhood (6 blocks)	432	247	182	0.74 (0.56 - 0.98)*	0.84 (0.65 - 1.10)	199	88	100	1.14 (0.81 - 1.60)	1.31 (0.94 - 1.84)
Out of neighborhood ( > 6 blocks)	346	211	144	0.68 (0.48 - 0.96)*	0.85 (0.62 - 1.16)	161	83	75	0.90 (0.62 - 1.29)	1.04 (0.73 - 1.49)
Specific location,										
Sidewalk, street, curb	323	152	156	1.03 (0.75 - 1.41)	1.28 (0.94 - 1.73)	178	70	95	1.35 (0.92 - 1.97)	1.66 (1.14 - 2.43)**
Just outside private home	220	134	87	0.65 (0.45 - 0.93)*	0.74 (0.52 - 1.05)	97	49	45	0.93 (0.59 - 1.47)	1.04 (0.65 - 1.66)
Recreational	117	104	36	0.34 (0.17 - 0.70)**	0.30 (0.15 - 0.58)**	28	22	10	0.43 (0.15 - 1.20)	0.39 (0.14 - 1.10)
Parking place	91	53	39	0.73 (0.45 - 1.18)	0.79 (0.48 - 1.28)	47	25	21	0.86 (0.47 - 1.59)	0.92 (0.49 - 1.71)
Public transit	31	20	11	0.55 (0.19 - 1.59)	1.21 (0.40 - 3.65)	12	08	NC	0.54 (0.14 - 2.07)	0.76 (0.20 - 2.83)
**II. Activity at time of outdoor fall**										
Walking	356	190	158	0.83 (0.61 - 1.13)	0.98 (0.74 - 1.30)	165	72	83	1.15 (0.80 - 1.66)	1.42 (1.00 - 2.02)
Vigorous activity	109	92	35	0.38 (0.18 - 0.81)*	0.42 (0.21 - 0.84)*	32	24	11	0.45 (0.18 - 1.11)	0.51 (0.20 - 1.27)
Going up or down stairs	107	55	49	0.90 (0.56 - 1.42)	1.01 (0.63 - 1.61)	63	30	31	1.00 (0.57 - 1.76)	1.09 (0.62 - 1.93)
Single step/curb	50	25	24	0.94 (0.51 - 1.73)	1.07 (0.57 - 2.00)	29	13	15	1.10 (0.51 - 2.38)	1.16 (0.53 - 2.56)
Gardening/lawn care	47	28	18	0.67 (0.32 - 1.38)	0.94 (0.44 - 1.98)	14	NC	09	3.41 (0.71 - 16.42)	4.28 (0.87 - 21.02)
Getting in/out of something	28	17	11	0.67 (0.31 - 1.43)	0.66 (0.30 - 1.43)	13	08	05	0.67 (0.23 - 2.01)	0.54 (0.17 - 1.72)
Not moving	21	13	08	0.63 (0.25 - 1.59)	0.77 (0.31 - 1.90)	11	NC	06	1.53 (0.37 - 6.26)	1.93 (0.47 - 7.85)
Bending	15	11	05	0.50 (0.16 - 1.58)	0.44 (0.13 - 1.53)	7	04	NC	0.77 (0.17 - 3.44)	0.72 (0.14 - 3.59)
Other	37	20	17	0.84 (0.42 - 1.69)	1.09 (0.54 - 2.19)	24	10	12	1.16 (0.49 - 2.75)	1.63 (0.67 - 3.98)
**III. Outdoor environment**										
Surface conditions										
Dry	494	259	221	0.86 (0.65 - 1.12)	1.01 (0.79 - 1.30)	275	129	134	1.04 (0.76 - 1.41)	1.17 (0.87 - 1.58)
Snowy/icy	199	140	77	0.55 (0.36 - 0.86)**	0.57 (0.38 - 0.87)**	54	24	28	1.16 (0.63 - 2.12)	1.34 (0.73 - 2.43)
Wet	71	43	28	0.65 (0.36 - 1.16)	0.86 (0.47 - 1.55)	32	18	14	0.74 (0.35 - 1.56)	0.92 (0.42 - 2.03)
Slipped or tripped	518	291	223	0.77 (0.58 - 1.02)	0.85 (0.66 - 1.11)	231	101	117	1.17 (0.83 - 1.64)	1.30 (0.94 - 1.81)
Poor lighting	49	22	25	1.15 (0.57 - 2.32)	1.30 (0.65 - 2.60)	24	NC	14	1.73 (0.65 - 4.61)	1.93 (0.72 - 5.12)
**Total indoor falls**	894	428	446	1.05 (0.82 - 1.34)	1.18 (0.94 - 1.48)	351	107	209	1.98 (1.44 - 2.72)**	1.96 (1.44 - 2.68)**
**I. Place of indoor fall**										
In own home	726	355	363	1.03 (0.79 - 1.35)	1.20 (0.93 - 1.54)	263	86	155	1.84 (1.30 - 2.59)**	1.84 (1.31 - 2.58)**
Living/family room	183	96	88	0.93 (0.62 - 1.39)	1.03 (0.70 - 1.52)	48	23	27	1.44 (0.70 - 2.97)	1.42 (0.70 - 2.85)
Bedroom	156	59	85	1.44 (0.91 - 2.28)	1.55 (0.98 - 2.46)	64	20	38	1.90 (0.99 - 3.64)	1.89 (0.97 - 3.68)
Stairs in home	99	66	37	0.57 (0.34 - 0.95)*	0.51 (0.30 - 0.86)*	38	13	21	1.61 (0.76 - 3.41)	1.53 (0.72 - 3.22)
In home, other/don’t know	95	60	40	0.68 (0.35 - 1.31)	0.94 (0.49 - 1.81)	19	11	09	0.96 (0.36 - 2.57)	0.92 (0.34 - 2.47)
Kitchen/dining room	87	29	51	1.88 (1.04 - 3.40)*	2.72 (1.42 - 5.22)**	39	04	27	6.83 (2.05 - 22.79)**	6.37 (1.91 - 21.26)**
Hall/lobby/doorway	60	24	33	1.37 (0.73 - 2.57)	1.37 (0.73 - 2.57)	29	09	17	1.82 (0.75 - 4.44)	1.78 (0.72 - 4.36)
Bathroom	46	16	26	1.63 (0.79 - 3.37)	2.09 (0.94 - 4.63)	26	08	15	1.92 (0.74 - 5.00)	3.45 (1.01 - 11.73)*
In another home	37	12	21	1.80 (0.84 - 3.87)	1.73 (0.81 - 3.71)	18	03	12	4.65 (1.05 - 20.66)*	4.43 (1.00 - 19.60)
Inside another building	131	62	63	1.02 (0.66 - 1.57)	1.02 (0.66 - 1.58)	70	19	42	2.29 (1.18 - 4.44)*	2.23 (1.13 - 4.40)*
**II. Activity at time of indoor fall**										
Walking	324	147	172	1.20 (0.84 - 1.70)	1.46 (1.04 - 2.06)*	129	32	82	2.47 (1.50 - 4.06)**	2.51 (1.52 - 4.16)**
Vigorous activity	34	NC	20	1.78 (0.74 - 4.31)	1.78 (0.71 - 4.44)	13	04	08	1.88 (0.49 - 7.27)	1.73 (0.44 - 6.74)
Going up or down stairs	132	81	53	0.66 (0.43 - 1.02)	0.63 (0.40 - 0.97)*	59	21	33	1.55 (0.85 - 2.82)	1.48 (0.82 - 2.69)
Single step/curb	4	02	02	0.61 (0.05 - 7.12)	1.03 (0.05 - 19.37)	0	0	0	--	--
Household tasks	44	08	29	3.68 (1.50 - 8.98)**	3.48 (1.41 - 8.55)**	19	03	13	4.91 (1.13 - 21.26)**	NC
Getting in/out of something	135	55	70	1.29 (0.81 - 2.04)	1.51 (0.94 - 2.41)	55	16	33	2.04 (1.02 - 4.08)*	2.59 (1.21 - 5.56)*
Not moving	94	49	45	0.93 (0.55 - 1.57)	0.95 (0.57 - 1.57)	35	14	19	1.42 (0.64 - 3.18)	1.30 (0.59 - 2.90)
Bending	35	17	17	0.95 (0.43 - 2.10)	0.92 (0.40 - 2.10)	15	05	08	1.52 (0.40 - 5.76)	1.32 (0.34 - 5.12)
Other	68	31	34	1.09 (0.59 - 2.00)	1.09 (0.59 - 2.03)	26	08	15	1.86 (0.69 - 4.98)	1.73 (0.64 - 4.69)
**III. Indoor environment**										
Surface conditions										
Dry	858	408	431	1.06 (0.82 - 1.37)	1.20 (0.95 - 1.51)	334	102	200	2.01 (1.45 - 2.79)**	1.98 (1.44 - 2.74)**
Wet	31	17	14	0.80 (0.39 - 1.64)	0.82 (0.40 - 1.71)	15	07	08	1.16 (0.38 - 3.59)	1.38 (0.40 - 4.69)
Slipped or tripped	384	157	203	1.29 (0.96 - 1.74)	1.32 (0.99 - 1.75)	159	41	97	2.35 (1.54 - 3.59)**	2.34 (1.54 - 3.57)**
Light conditions too poor to see	69	24	39	1.63 (0.92 - 2.87)	1.56 (0.89 - 2.76)	36	07	NC	NC	NC

Sample population 267 men and 467 women with a total of 1680 falls with indoor/outdoor location information, and a total follow-up time of 2066.5 person years.

All rates and rate ratios estimated by negative binomial regression.

*Abbreviations*: RR Rate Ratio, ratio of women rate to men rate. NC Regression model does not converge (too few observations).

^#^Number of falls for men and women combined. *p-value <0.05 **p-value <0.01.

†Adjusted for a risk score composite of all factors in Table 1: age, education, race, body mass index, physical activity, blocks walked per week, outdoor walking type, balance, chair-stand ability, gait speed, activities of daily living, short physical performance battery, reduced activity due to illness in past year, poor vision, bodily pain, number of comorbidities, self-rated health, peripheral neuropathy, foot pain, knee osteoarthritis, depression, number of medications, use of psychotropic medication, MMSE, Trails-B score, number of falls in year before baseline and falls efficacy.

Adjustment for composite risk scores made only marginal difference in the magnitude of most rate ratios. In general, the women/men ratio in fall rates was slightly increased for most types of falls by the adjustment. For example, after adjustment the women-to-men rate ratio for all outdoor falls increased from 0.72 (0.56-0.92) to 0.84 (0.67 -1.05), and the rate ratio for all indoor falls increased from 1.05 (0.82-1.34) to 1.18 (0.94 -1.48), (Table 2).

### Rates of injurious falls

Injurious outdoor fall rates were equivalent between sexes despite the greater fall rates in men (Table 2). Women had significantly higher rates of injurious indoor falls overall (women-to-men RR: 1.98, 1.44-2.72), in the kitchen (6.83, 2.05-22.79), in their own home (1.84, 1.30–2.53) and within another residential home (4.65, 1.05-20.66) or building (2.29, 1.18-4.44) compared to men (Table 2). Daily activities associated with greater rates of injurious indoor falls in women included walking (2.47, 1.50-4.06), performing household tasks (4.91, 1.13-21.26) and getting in or out of something such as the bath tub (2.04, 1.02-4.08). The greater tendency for injury in women can be seen in their significantly higher rates of injurious indoor falls on dry surfaces (2.01, 1.45-2.79) or from slipping or tripping on something (2.35, 1.54-3.59) (Table 2). In all indoor circumstances combined, women had a significantly greater rate of injurious falls compared to men (1.98, 1.44-2.72), and their rate of hospital admissions due to a fall was nearly twice that of men (1.90, 0.92-3.94) (Table 3).

**Table 3:** Sex-specific rates of injurious falls (per 100 person-years) and women-to-men rate ratios (RR) with 95% confidence intervals, by place of falling and injury type and severity

	**Men**	**Women**	**Crude RR**	**Adjusted**^**# **^**RR**
**Outdoor falls**				
Any injury	17.4 (14.2 - 21.3)	17.6 (14.7 - 21.0)	1.01 (0.77 - 1.33)	1.15 (0.88 - 1.51)
Fracture	1.3 (0.7 - 2.5)	1.9 (1.3 - 2.8)	1.44 (0.69 - 3.01)	1.77 (0.85 - 3.68)
Other serious injury	2.1 (1.2 - 3.6)	2.1 (1.4 - 3.1)	0.99 (0.51 - 1.93)	1.12 (0.58 - 2.18)
Hospital admission	0.8 (0.4 - 1.8)	0.9 (0.5 - 1.7)	1.14 (0.41 - 3.20)	1.08 (0.37 - 3.17)
**Indoor falls**				
Any injury	10.7 (8.0 - 14.3)	20.9 (17.8 - 24.6)	1.98 (1.44 - 2.72)**	1.97 (1.44 - 2.68)**
Fracture	0.8 (0.2 - 2.8)	2.9 (2.0 - 4.2)	4.28 (1.58 - 11.62)**	4.68 (1.52 - 14.4)**
Other serious injury	1.6 (0.9 - 2.8)	3.6 (2.6 - 5.0)	2.24 (1.14 - 4.42)*	2.48 (1.20 - 5.13)*
Hospital admission	1.6 (0.7 - 3.6)	3.0 (2.1 - 4.2)	1.90 (0.92 - 3.94)	1.71 (0.81 - 3.60)

“Any injury” includes any positive response to the query “Did you hurt yourself in any way when you fell?”

“Other serious injury” includes sprains; pulled or torn muscles, tendons or ligaments; dislocated joints; and concussions.

All rates and rate ratios estimated by negative binomial regression.

*p-value <0.05, ** *p-value <0.01.*

^#^Rate ratios adjusted for a combined risk score composed of the characteristics in Table 1: age, education, race, body mass index, physical activity, blocks walked per week, outdoor walking type, balance, chair-stand ability, gait speed, activities of daily living, short physical performance battery, reduced activity due to illness in past year, poor vision, bodily pain, number of comorbidities, self-rated health, peripheral neuropathy, foot pain, knee osteoarthritis, depression, number of medications, use of psychotropic medication, MMSE, Trails-B score, number of falls in year before baseline and falls efficacy.

As with fall rates, adjustment for composite risk scores made little difference in the rate ratios of injurious falls. The rate ratio for injurious falls on sidewalks, streets and curbs increased 23% after adjustment, from 1.35 (0.92-1.97) to 1.66 (1.14 -2.43) (Table 3), but this was the exception. Further investigation of the factors making up the composite risk score showed that the difference in the number of blocks walked accounted for most of the confounding effect seen here, i.e., if women walked as many blocks as men do, their relative injury rate on sidewalks, streets, and curbs would be even greater.

## Discussion

Men and women tend to differ in where and how they spend their time as well as in many physical characteristics, and these differences are reflected in the circumstances of their falls and resulting injuries. This study reports novel findings on significant sex differences in rates of falling in various indoor and outdoor places and when performing select activities. Such information is critical to the development of future falls prevention programs that account for sex differences in behaviors, space use and activity patterns. Men had significantly greater rates of outdoor falls in specific recreational locations and while engaging in vigorous activities. Despite near equal indoor fall rates between men and women, women had significantly higher rates of injurious indoor falls. These results suggest that fall injury prevention strategies need to consider sex differences in activity patterns, space use and fall-related behaviors. The analysis also demonstrates the relative importance of various indoor and outdoor places for fall injury preventions.

As with our previous work [13,23] this study shows numerous differences in rates and consequences of indoor and outdoor falls, adding evidence of sex differences in fall rates by circumstance and injury potential. In accordance with Li et al [16] we found in an older population that men reported a significantly greater rate of outdoor falls than women. Increased fall rates for men were notable in specific locations of recreation such as at golf courses, forests or parks. These findings are in accordance with previous studies showing higher leisure time activity is associated with falling outdoors [16,27,28]. Furthermore, men who fell outdoors reported greater rates of falling during vigorous activities such as hiking, tennis, and jogging compared to women. In contrast, women fell equally as often as men outside on sidewalks, streets and curbs.

Although equivalent in age, the men and women in this study had numerous differences in physical and behavioral characteristics. Men were significantly more educated and reported more recreational outdoor activities compared to women. Furthermore, women reported higher levels of bodily pain and use of psychotropic medication compared to men, which may have resulted in women scoring significant lower on a number of functional tests (gait, balance, SPPB) compared to men. Although differences in characteristics existed, sex differences in fall rates were not substantially explained by these factors when adjustments were added to the analysis.

Many of the gender differences in fall rates are likely due to differences in their exposure time. This study was not designed specifically to measure time spent in various activities, but the differences observed with the components of the PASE questionnaire (occupational physical activity, light housework and garden chores) may shed light on the degree of gender difference. For example, men spent significantly more time overall in recreational activities. Men spent on average 0.5 hours per day in light recreation as compared to 0.3 hours for women, 0.4 vs. 0.3 hours per day in moderate recreation, and 0.4 vs. 0.3 hours per day in vigorous activities. However, a large proportion of participants of both sexes claimed no recreational activities in the past week (61.6% of men vs. 68.3% of women). Women reported spending significantly more time doing light household chores compared to men, corresponding to greater fall rates in specific indoor rooms.

Consistent with prior studies [11,14], 53% of falls were reported in indoor locations. Despite men having greater rates of falls outdoors, the total indoor fall rates were nearly equal between the sexes. Similar to previous research [11,15] indoor falls were most frequently reported in the living room, bedroom and kitchen. Furthermore, women reported significantly greater rates for falling in the kitchen and while performing household tasks. Regarding exposures that might contribute these differences, participants were asked only if they had performed any light housework such as dusting or washing dishes during the past seven days, and 84.2% of men answered yes vs. 98.0% of women (p < 0.001). Future studies should consider collecting detailed information of the time spent in various indoor and outdoor activities to further explain the differences in fall rates among older adults due to exposure times in these activities.

Among all activities, walking is reported as the predominant form of physical activity for older adults. In the current study 45% of outdoor falls occurred in men and women while walking, which is consistent with previous studies showing proportions of falls due to walking ranging from 36% to 63% [12,14,29-31]. Although it is evident in this study that men walked significantly more city blocks per week than women there were no significant sex differences in the rates of falling outdoors while walking. Nonetheless, high proportions of outdoor falls due to walking may develop a barrier to physical activity in those prone to falling thus leading to decreased independence and mobility, and increased numbers of older adults becoming homebound [16,25].

Due to the lack of well-developed methods for evaluating outdoor environmental hazards for falls it is difficult to determine which outdoor factors are most likely to increase fall rates in older adults [15,32]. However, it is clear from these findings that men when compared to women were significantly more likely to fall outdoors in snowy or icy environmental conditions. In the present study, outdoor fall rates due to environmental features such as wet surfaces, and tripping or slipping appeared higher in men. Future falls studies may consider further investigation into the environmental hazards for falling outdoors by studying the built environment. Better access to spatial statistics, geographic information and the public databases, when combined with data on fall circumstances will allow researchers to determine whether particular features and conditions of sidewalks, streets, parks and other areas in which older adults travel may be associated with their rates of falling [16].

Despite nearly equal indoor fall rates, the *injurious* indoor fall rate in women was significantly higher than that of men, especially in the kitchen of their own home, as well as inside other buildings and other homes. While previous studies have suggested that any given indoor fall carries less risk of injury than an outdoor fall [33], we found women had a higher absolute rate of injurious indoor falls than injurious outdoor falls. Injury prevention strategies should thus continue in women to target the indoor environment. Compared to men, women had twice the rate of experiencing an injurious fall indoors and four times the risk of suffering a fracture. The increased rate of fracture in women may be due to the increased prevalence of osteoporosis in women compared to men making them more susceptible to fracture [12]. Outdoors, where men had higher rates of falls, the rates of injurious falls overall were equivalent for the sexes. Thus, in both indoor and outdoor falls, women appear to have a greater propensity for injury when a fall occurs. By far the greatest rate of injurious outdoor falls for both sexes occurred on sidewalks, streets, and curbs, suggesting that injury-prevention strategies would benefit from studying the specific hazards leading to falls in these locations.

This analysis has several strengths and limitations. The strengths include the prospective longitudinal follow-up, the relatively large sample size, a sample well representing the underlying community-dwelling older population, and the well-ascertained daily fall documentation using monthly calendars [34]. The limitations, however, of this study include: firstly, the results from this study may not be generalized to other populations as this cohort was drawn from only one area (Boston, MA). Longitudinal studies in other geographic regions are needed to confirm our findings. Secondly, individuals were not asked to report the amount of time they spent indoors and outdoors, and in specific locations. Although we had some inexact indicators of time spent in specific activities, adjusting for these in our regressions did not change the risk ratios enough to “explain” the sex difference in fall rates. We expect that more exact activity logs would help clarify why the rate and circumstance of outdoor falls were different in men and women. Previous indications have shown that average American men will spend approximately 78 minutes per day outdoors which may seem potentially small. However, knowing the time spent outdoors will allow for the calculation of rates for falling accounting for time [35]. However it is important to indicate that, our primary purpose for this study was not to explain but rather to merely document these differences. Thirdly, the data collected on the occurrence of falls in this population was based on self-report, which is subject to recall inaccuracies. However in the current study a large number of circumstances of falls were examined using fall calendars as well as interviews to try to reduce the inaccuracies, providing reliable data on outdoor and indoor falls. Finally, despite the relatively large sample size, in some instances the category numbers were small and thus it was not possible to consider less common types of falls and injuries. It will be important to further examine the circumstances of injurious falls when larger numbers of these events have occurred.

## Conclusions

In conclusion this study shows that there are sex differences in rates of location- and activity-specific falls that are important to consider in future falls prevention programs. The sex differences may in part be attributable to differences in exposure time, with men spending more time outdoors in recreational locations and performing vigorous activity. Women had greater risk for fall injuries than men especially when performing indoor activities. These findings highlight a need for focused falls prevention strategies, which consider differences in how older men and women spend time and use space, and how they interact with the indoor and outdoor environment.

## Competing interests

The authors declare that they have no competing interests.

## Authors’ contributions

WL conceived of the study and obtained the funding. WL, RD and EPG formulated the statistical analysis plan. EPG carried out the analysis. RD, EPG and WL interpreted the statistical results. RD drafted the manuscript. MTH, SL and LL are the investigators of the MOBILIZE Boston Study which collected the data. All authors contributed to the manuscript writing. All authors read and approved the final manuscript.

## Pre-publication history

The pre-publication history for this paper can be accessed here:

http://www.biomedcentral.com/1471-2318/13/133/prepub
